# Perceptions of end-of-life care quality among bereaved closest contacts of community-dwelling older Australians: a cross-sectional survey of the ASPREE cohort

**DOI:** 10.21203/rs.3.rs-9879018/v1

**Published:** 2026-06-17

**Authors:** Zoe M. Keon-Cohen, Cammie Tran, Daryl Jones, Pamela Y. L. Wong, Catherine Robb, Jane Banaszak-Holl, John J. McNeil

**Affiliations:** Monash University; Monash University; Monash University; Monash University; Monash University; University of Alabama at Birmingham; Monash University

**Keywords:** End-of-life care, Advance care planning, Aged, Quality of health care, Terminal care, Bereavement, Patient-reported outcome, VOICES survey, Australia

## Abstract

**Background:**

High-quality end-of-life care (EOLC) is a recognised health and human-rights priority, yet Australian evidence largely derives from administrative data and cancer-specific, facility-based samples. Little is known about the quality of EOLC experienced by community-dwelling older adults across the full range of care settings. We assessed perceived EOLC quality and its socio-demographic and clinical correlates in this group.

**Methods:**

We conducted a cross-sectional survey of the closest contacts of deceased participants in the ASPirin in Reducing Events in the Elderly (ASPREE) study, a cohort of initially healthy, community-dwelling adults aged 70 years and older. The 32-item instrument, adapted from the UK Views of Informal Carers — Evaluation of Services, Short Form (VOICES-SF) and refined through consumer and expert review, captured overall EOLC quality and five domains (pain control, symptom control, communication, emotional peace, and support) during the final month of life. Responses were linked to prospectively collected ASPREE data. Associations were examined using Pearson chi-square or Fisher exact tests.

**Results:**

Of 432 invitations, 342 responded (79% response rate) and 341 were analysed. Overall EOLC in the last month of life was rated very good or excellent by 84% of respondents and poor or very poor by 6%. Ratings were similarly high for pain control (80%), symptom control (84%), communication (79%), emotional peace (79%), and support (81%). Overall EOLC quality did not differ significantly by age, sex, rurality, socio-economic position (IRSAD), education, place of death, cancer status, illness trajectory, or advance care planning documentation. Domain-specific differences were limited: closest contacts of younger decedents more often reported poorer communication (p = 0.009), female decedents less often achieved a sense of peace (p = 0.01), and decedents with ≤ 12 years of education were rated as better supported (p = 0.05).

**Conclusions:**

In this community-dwelling cohort of older Australians, perceived EOLC quality was uniformly high and showed no socio-economic, geographic, or diagnostic gradient, indicating that equitable, high-quality EOLC is achievable in a well-resourced population. These data provide a community-based benchmark against which disparities in less advantaged groups can be measured, and identify communication with younger families as a target for improvement.

## Background

The nature of end-of-life care (EOLC) is evolving in response to medical advances, an ageing population with increasing comorbidity, and rising community expectations of high-quality end-of-life care as a human right [1, 2]. For more than a decade the need to improve end-of-life care has been recognised by the World Health Assembly, national legislation, policies, and guidelines [3]. An Australian parliamentary inquiry found mixed views on the current quality of EOLC [4]. Existing knowledge has relied largely on administrative data, small surveys, and coronial reports, which do not capture the experiences of patients and carers.

In Australia, the Royal Commission into Aged Care Quality and Safety (2021) [5], the Aged Care Act 2024 (Cth) [6], and the Australian Commission on Safety and Quality in Health Care [7] support these changes. Several tools have been proposed to measure EOLC quality across care settings, although none has yet been accepted as a gold standard [8]. Widely accepted elements of quality EOLC include pain and symptom control, peace of mind, preparedness for death, and communication with care providers; additional elements include maintaining physical independence, a sense of dignity, the accessibility of family and friends, meeting emotional and spiritual needs, and providing bereavement support for caregivers [9, 10].

The United Kingdom Views of Informal Carers — Evaluation of Services, Short Form (VOICES-SF) questionnaire and its derivatives were first implemented in England in 2011 to capture carers’ experiences of EOLC quality for patients across a broad range of settings, including hospitals, hospices, care homes, and home [11, 12, 13]. Early implementations were limited largely to patients with cancer and focused on the location of death as a proxy for quality [14, 15]. Evaluation of EOLC has since become routine in the UK, supported by NICE guidance [16], but is rarely undertaken in the general Australian community.

The ASPirin in Reducing Events in the Elderly (ASPREE) study was a US/Australian randomised, placebo-controlled trial of low-dose aspirin that enrolled 19,114 initially healthy, community-dwelling individuals aged 70 years and older [17]. At recruitment, participants were free of evident cardiovascular disease, dementia, independence-limiting physical disability, or any chronic illness likely to limit survival to less than five years, and were recruited through general practitioners in both urban and rural settings. Because of its long-term engagement with participants (and frequently their closest contacts) and the extensive information collected before terminal illness, ASPREE offers a unique opportunity to examine the circumstances surrounding participants’ deaths [18].

Our aim was to obtain perceptions of EOLC quality from the closest contacts of recently deceased ASPREE participants across five domains — pain control, symptom control, communication, emotional peace, and support — and to examine associations between these domains and clinical and demographic characteristics.

## Methods

### Study aim and objectives

This study aimed to assess the quality and nature of EOLC in the final month of life among community-dwelling older Australians by surveying the closest contacts of deceased ASPREE participants, and to examine associations across five domains of EOLC: pain control, symptom control, communication, emotional peace, and support. We hypothesised that, given the relatively advantaged profile of ASPREE participants, overall EOLC quality would be high and that no significant associations would be observed between overall EOLC quality and socio-economic, geographic, or clinical characteristics. We report the study in accordance with the STROBE (Strengthening the Reporting of Observational Studies in Epidemiology) reporting guideline for cross-sectional studies.

### Design, setting, and participants

This was a cross-sectional, population-based bereavement survey nested within the ASPREE prospective cohort study. We surveyed the closest contacts of deceased ASPREE participants; each participant had nominated up to two close contacts. A pilot study was completed between September 2019 and September 2020, followed by the remaining data collection through to December 2022. A total of 432 closest contacts were invited to complete the ASPREE quality-of-EOLC survey, identified in the ASPREE study database as the nominated primary contact for deceased participants. Socio-demographic and health data were sourced from the ASPREE database.

### Development of the survey instrument

An expert committee of ASPREE investigators, medical specialists, and ASPREE trial participants reviewed and adapted questions derived primarily from the 2017 and 2019 revised short-form UK VOICES-SF questionnaire to align with current Australian health practice. The instrument was designed for appropriateness, clarity, and sensitivity, using brevity and predominantly frequency-based response options to maximise the response rate and minimise the risk of distress. The committee recommended excluding one question regarding resuscitation preferences. In addition to overall EOLC quality, the survey assessed five domains, as perceived by bereaved relatives: (1) pain control, (2) symptom control, (3) support, (4) quality of communication, and (5) emotional peace. Three iterations were developed and revised before ethics approval. Two versions of the survey were used; the second added one question following an initial pilot survey of 50 participants. The final survey comprised 32 questions (30 multiple-choice and 2 short-answer; Additional file 1). The study’s affiliation with Monash University and ASPREE was stated in the mailed invitation letter [12] (Additional file 2).

### Eligibility and survey distribution

**Figure 1.**
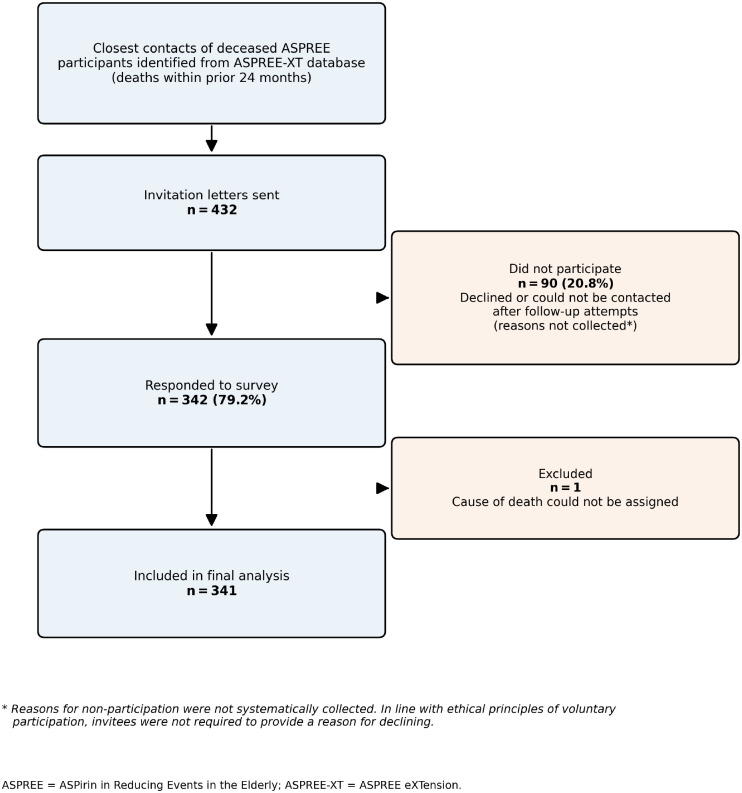
Participant flow through the ASPREE Closest Contact Survey of End-of-Life Care (STROBE-style flow diagram): 432 closest contacts invited → 342 responded (79%) → 1 excluded (cause of death unassignable) → 341 analysed.

Deceased ASPREE participants were eligible if they died within 24 months of sub-study screening and had one or more closest contacts listed in the ASPREE-XT database. An invitation letter, including an option to opt out, was sent to potential respondents. Participants were excluded if they were non-English-speaking, had withdrawn from ASPREE or elected to provide follow-up only via medical records, did not respond to three follow-up calls, or exhibited distress during the interview. Data were collected via telephone interview, email, or postal survey. A study flow diagram is provided in Fig. 1.

### Outcomes

The primary outcome was the overall perceived quality of EOLC. Secondary outcomes were the perceived quality of pain control, symptom control, emotional peace, communication about treatment and side effects, level of support at home, and the presence or absence of advance care planning (ACP) documentation.

### Data classification and statistical analysis

Socio-demographic and health data were sourced from the ASPREE database, including age, sex, postcode-derived rurality (regional vs metropolitan), education (≤ 12 vs > 12 years), the Index of Relative Socio-economic Advantage and Disadvantage (IRSAD), place of death (home vs other), adjudicated cause of death (classified by illness trajectory as rapid or chronic), and cancer status. Deaths were classified into sudden, rapid, or chronic trajectories per ASPREE adjudication (Additional file 3); sudden deaths were excluded owing to limited opportunity for EOLC intervention. Covariate data derived from the ASPREE database were available for the majority of participants; missing covariate values were handled by complete-case analysis. Responses were categorised as positive (excellent or very good), neutral (satisfactory), or negative (poor or very poor), with “don’t know”/“not relevant” reported separately. The numbers excluded from each domain denominator as “don’t know”/“not applicable” are reported in Additional files 4–8.

No formal sample size calculation was performed; all eligible closest contacts in the ASPREE-XT database were invited to participate. Descriptive statistics were generated in REDCap; analyses were conducted in Stata v17.0 (StataCorp LLC, College Station, TX, USA). Continuous variables were dichotomised at the sample median to maximise statistical power in the absence of established clinical thresholds: age (> 77 vs ≤ 77 years), education (> 12 vs ≤ 12 years), and IRSAD (> 6 vs ≤ 6); remaining variables were categorised as sex, rurality (metropolitan vs regional), place of death (home vs other), ACP documentation (present vs absent), illness trajectory (rapid vs chronic), and cancer diagnosis (present vs absent). Categorical data were analysed using the Pearson chi-square test, except for illness trajectory and, where applicable, place of death, which were analysed using the two-sided Fisher exact test. A two-sided p-value < 0.05 was considered statistically significant. Confidence intervals for the primary percentages were calculated using the Clopper-Pearson exact method from the denominators reported in Additional files 4–8. No sensitivity analyses or confounding adjustments were performed. We acknowledge the use of Microsoft Copilot for literature searching and for proofreading and editing of the manuscript.

## Results

### Respondents

**Figure 2:**
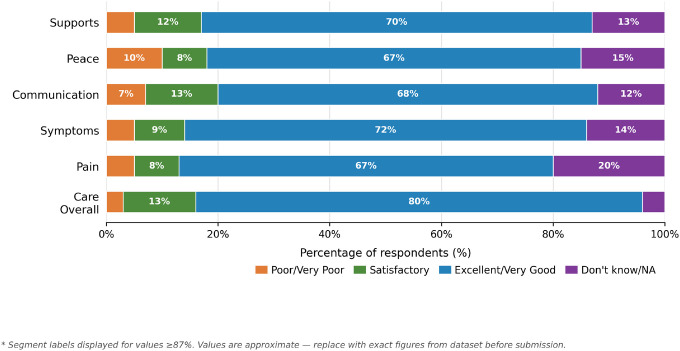
Distribution of responses across the six EOLC quality indicators (overall care, pain, symptom control, communication, emotional peace, and support), shown as the percentage of respondents rating each indicator poor/very poor, satisfactory, excellent/very good, or don’t know/not applicable.

Of 432 invitation letters sent, 342 closest contacts completed the survey (79.2% response rate; 342/432) and 90 (20.8%) either declined participation or could not be contacted after follow-up; reasons for non-participation were not collected, as invitees were not required to provide a reason for declining (Figure 1). One respondent was excluded because a cause of death could not be assigned, leaving 341 in the final analysis. The achieved sample provides 95% confidence intervals of approximately ±5 percentage points for the primary outcome. No respondent reported distress from the interview. Telephone interview was the primary mode of data collection (84%). The distribution and counts of Likert responses across all six EOLC indicators are illustrated in Figure 2.

### Decedents

Decedents had a mean age at death of 78 years; 48% were female and 78% were born in Australia. All were living at home at ASPREE enrolment, of whom 38% lived alone. Among the closest contacts surveyed, 64% were the decedent’s child, 20% a partner, and 4% a sibling; 63% had been in daily contact with the decedent in the months before death. Place of death was distributed across home (≈21% of valid responses), hospital wards (38%), supported accommodation (30%), hospice (5%), and emergency or intensive care units (2%). Additional demographic and clinical characteristics are shown in Table 1.

### Overall quality of EOLC

Overall EOLC during the last month of life was rated very good or excellent by 84% of respondents (95% CI 79–88%) and poor or very poor by 6% (Table 2; Figure 2). Of the 65 valid responses concerning participants who died at home (21%), 86% rated overall care as very good or excellent and 3% as poor or very poor. Overall EOLC ratings did not differ significantly by ACP status, cancer diagnosis, education, IRSAD score, rurality, age, sex, illness trajectory, or place of death (all p > 0.05; Table 2).

### Five domains of EOLC

Pain control was rated excellent or very good for 80% of decedents (95% CI 74–85%; n = 226), with no socio-demographic or clinical variable significantly associated with poorer pain control (Additional file 4). Respondents who reported no pain, “don’t know”, or “not relevant” (33%, n = 113) were excluded from this analysis. Symptom control was rated excellent or very good for 84% of decedents, with no significant associations (Additional file 8). Communication about illness, treatment, and side effects was rated very good or excellent by approximately 79% of respondents (95% CI 73–83%; n = 282); closest contacts of younger decedents more often reported poorer communication than those of older decedents (p = 0.009; Additional file 5). Emotional peace was reported as “very much” or “mostly” present for about 79% of decedents (95% CI 74–84%; n = 256), with female decedents significantly more likely to be rated as having a poor or very poor sense of peace (p = 0.01; Additional file 6). Perceived support at home was generally high (81%); decedents with ≤12 years of education were more likely to be rated as better supported than those with higher education (p = 0.05), with no other significant associations (Additional file 7). Forty-nine percent of respondents reported documented ACP at death; ACP status was not significantly associated with overall perceived EOLC (Table 2).

## Discussion

### Summary of findings

This study examined closest contacts’ perceptions of EOLC for deceased ASPREE participants, focusing on overall quality and five domains: pain control, symptom control, communication, emotional peace, and support. Most respondents (84%) rated overall EOLC as very good or excellent, with similarly high proportions reporting good pain and symptom control and a sense of peace, and roughly four in five reporting good communication and support. The most notable finding was the absence of socio-economic, geographic, diagnostic, or care-setting gradients in overall EOLC quality: ratings varied minimally across rurality, socio-economic status, cancer status, illness trajectory, place of death, and ACP documentation. Domain-specific differences were confined to three signals — poorer communication reported for younger decedents, a lower sense of peace for female decedents, and better-rated support for decedents with lower education.

### Comparison with previous studies

The pattern of high overall satisfaction and low rates of poor care, particularly for home- and hospice-based care, is consistent with international, UK, and European community-based studies using the VOICES and VOICES-SF surveys [11, 13, 19]. Our rate of home deaths (≈ 21%) is similar to that reported in the UK (13%) [13], Europe [20], Canada, and the USA [21], but lower than in Taiwan (36%) [22], where more home-based EOLC services may be available for patients with cancer. Whether EOLC quality is genuinely better when death occurs at home remains unclear. The finding that closest contacts of younger participants more often reported poorer communication is consistent with bereaved-relative survey findings that preferences for place of death and the degree of shared decision-making vary with patient age and family involvement [23].

Other Australian studies have demonstrated inequities affecting vulnerable populations, such as those from migrant backgrounds [24], living with mental illness [25], or in rural or remote areas [26], and difficulty accessing well-timed specialist palliative care [27]; a previous study of patients in aged-care facilities reported that only about half received appropriate EOLC [28]. Studies in specific care settings have described delayed recognition of dying and late goals-of-care or palliative-care referrals when benchmarked against Australian national EOLC standards [7], and studies of rural and remote residents have reported less access to community palliative care and greater barriers to psychosocial and bereavement support than for metropolitan counterparts [29, 30, 31].

In contrast to these population-based studies, we observed no socio-economic or care-based differences indicating poorer EOLC quality in the ASPREE cohort, most plausibly reflecting a less vulnerable, healthier, and more advantaged population. Our findings complement evidence that cancer-related EOLC in Australia is generally high quality, supported by a robust health system, strong urban access to palliative services, and established care standards. We found no evidence that participants with a chronic trajectory received different EOLC from those with a more rapid trajectory.

### Interpretation: equitable, high-quality EOLC as the headline

The principal contribution of this study is positive. In a community-dwelling cohort spanning regional and metropolitan settings and both publicly and privately funded care, high-quality EOLC was reported uniformly and without the socio-economic gradient that characterises much of the EOLC literature. This demonstrates that equitable, high-quality EOLC is attainable within a well-resourced population, and it establishes a community-based reference standard against which the experiences of less advantaged groups can be benchmarked.

### Strengths and limitations

This is one of few studies to examine Australians’ perceptions of EOLC across a diverse cohort of community-based decedents in regional and metropolitan areas receiving privately or publicly funded care. It draws on a large, well-characterised cohort of initially healthy older adults with prospectively collected socio-demographic and clinical data, enabling linkage of closest-contact–reported EOLC experiences to information gathered before terminal illness. The survey instrument was adapted from the validated VOICES questionnaire used in the UK, Europe, and Canada, and was refined through multiple iterations with consumer input to maximise participation and minimise distress. The 79% response rate is high for a bereavement survey.

Several limitations apply. The modest sample size limits power for subgroup analyses, particularly among more disadvantaged populations in whom inadequate EOLC may be more prevalent; the domain-specific associations should therefore be regarded as hypothesis-generating. ASPREE participants were healthier, better educated, and more socio-economically advantaged than the general older Australian population, introducing healthy-volunteer and positive-selection biases that likely shift outcomes toward higher-quality care and limit generalisability to frailer or institutionalised populations. The direction of this bias is consistently toward overestimation of EOLC quality; its magnitude is difficult to quantify precisely, but comparative Australian evidence suggests that rates of poor or very poor care may be substantially higher in less selected populations. The survey did not capture several recognised dimensions of EOLC quality, including care intensity in the final month of life, coordination across services and settings, cultural safety, spiritual care, or bereavement support. Finally, perception-based responses and recall bias from closest contacts are unavoidable in this setting and may reflect respondents’ own expectations and experiences as carers rather than objective, contemporaneous, patient-reported outcomes.

### Implications for clinicians and policymakers

Recent legislative reform, national ACP initiatives, and shared decision-making frameworks have fostered greater public engagement with death as a natural life stage [32, 33]. For clinicians, the high ratings of overall care and of pain and symptom control are reassuring; however, the poorer communication reported by the closest contacts of younger decedents highlights the continuing need for clear, timely discussion of prognosis, goals of care, and likely illness trajectories. Earlier identification of patients approaching the end of life, clinician training, timely access to palliative care, proactive symptom assessment, and routine ACP across care settings remain central to aligning care with patient and family preferences and to reducing non-beneficial or burdensome intervention [34, 35].

For policymakers, ongoing system review can identify gaps, monitor equity, and evaluate interventions across hospitals, residential aged care, and community settings. An EOLC measurement tool could be extended to diverse settings — including First Nations peoples, culturally and linguistically diverse communities, and rural and remote populations — and could incorporate indicators of care intensity, coordination, cultural safety, and bereavement outcomes, to support equitable access to palliative care.

## Conclusions

In this large cohort of previously healthy, community-dwelling older Australians, closest contacts reported high overall EOLC quality, particularly for pain and symptom control, with few socio-demographic or clinical differences across strata. The absence of a socio-economic, geographic, or diagnostic gradient demonstrates that equitable, high-quality EOLC is achievable in a well-resourced population and provides a community benchmark for future comparison. Further research is needed to extend these standards to populations in greater need — including Australians from disadvantaged and culturally and linguistically diverse backgrounds — and to investigate the clinical factors influencing both the quality of, and equitable access to, timely palliative care and meaningful ACP for all Australians and their families.

## Supplementary Files

This is a list of supplementary files associated with this preprint. Click to download.

- AdditionalfilesBMCJune12026.docx

## Figures and Tables

**Table 1.** Demographic and clinical characteristics of deceased participants (n = 341)

Variable	Value
**Demographic**	
Age at randomisation, years	Median 77.6, IQR 73–81
Education >12 years	117 (35%)
Born overseas	75 (22%)
IRSAD*	Median 6, IQR 3–9
Ethnicity	337 (98%) White
**Clinical**	
Frailty	188 (55%)
Dementia	51 (15%)
**Relationship of closest contact**	
Partner	69 (20%)
Sibling	15 (4%)
Child	219 (64%)
Friend	14 (4%)
Neighbour	1 (0.3%)
Other	23 (7%)
**Contact frequency**	
Daily	214 (63%)
2–3 times/week	77 (22%)
Weekly	34 (10%)
Monthly	15 (5%)
**Living situation at enrolment** **	
Home alone	128 (38%)
Home with others	213 (62%)
Supported or nursing home	0

* IRSAD = Index of Relative Socio-economic Advantage and Disadvantage; sample median = 6.

** Living situation was reassessed annually for up to 9 years; supported accommodation rose from 0% to 16% over follow-up.

**Table 2.** Association between participant characteristics and overall EOLC quality Question 19: “Overall, how good was the overall care given [to the participant] during [his/her] final illness?”

Characteristic	Valid n	Poor/Very poor n (%)	Satisfactory n (%)	Excellent/Very good n (%)	Don’t know/NA	p-value
Overall	311	18 (6%)	33 (10%)	260 (84%)	22	–
Age >77	311	11 (61)	21 (63)	119 (46)	22	0.08
Age ≤77		7 (39)	12 (37)	141 (54)		
Female	311	9 (50)	19 (58)	131 (51)	22	0.74
Male		9 (50)	14 (42)	129 (49)		
IRSAD >6	309	11 (61)	20 (62)	144 (56)	22	0.70
IRSAD ≤6		7 (39)	12 (37)	115 (44)		
Metropolitan	309	10 (55)	13 (42)	119 (46)	22	0.60
Regional		8 (44)	19 (59)	140 (54)		
Education ≤12 y	311	4 (22)	13 (39)	98 (38)	22	0.40
Education >12 y		14 (78)	20 (61)	162 (62)		
Chronic trajectory	245	14 (88)	24 (96)	175 (86)	20	0.43
Rapid trajectory		2 (12)	1 (4)	29 (14)		
Cancer	311	8 (44)	16 (48)	132 (51)	20	0.85
Non-cancer		10 (56)	17 (52)	128 (49)		
ACP documented	256	13 (72)	18 (64)	128 (60)	20	0.24
No ACP		3 (18)	10 (36)	84 (40)		
Death at home	310	2 (11)	7 (21)	56 (22)	22	0.57
Death not at home		16 (89)	26 (79)	203 (78)		

Illness trajectory analysed with the two-sided Fisher exact test; all other variables with the Pearson chi-square test. P-values exclude “don’t know/NA” responses. No variable was significantly associated with overall EOLC quality at α = 0.05. ACP = advance care planning; IRSAD = Index of Relative Socio-economic Advantage and Disadvantage; NA = not applicable.

## Data Availability

The datasets generated and analysed during the current study are held within the ASPREE Safehaven data repository and are available from the ASPREE management committee on reasonable request, subject to data-governance approval.

## Abbreviations

ACP: advance care planning
ACSQHC: Australian Commission on Safety and Quality in Health Care
ASPREE: ASPirin in Reducing Events in the Elderly
ASPREE-XT: ASPREE eXTension
CI: confidence interval
EOLC: end of-life care
IQR: interquartile range
IRSAD: Index of Relative Socio-economic Advantage and Disadvantage
NA: not applicable
NICE: National Institute for Health and Care Excellence
REDCap: Research Electronic Data Capture
STROBE: Strengthening the Reporting of Observational Studies in Epidemiology
VOICES-SF: Views of Informal Carers – Evaluation of Services,Short Form
